# Study on the Shape Characteristics and the Allometry of *Phalaenopsis* Leaves for Greenhouse Management

**DOI:** 10.3390/plants12102031

**Published:** 2023-05-19

**Authors:** Jiunyuan Chen, Chiachung Chen

**Affiliations:** 1 Africa Industrial Research Center, National Chung Hsing University, 250 Kuokuang Road, Taichung 40227, Taiwan; 2 Department of Bio-Industrial Mechatronics Engineering, National Chung Hsing University, 250 Kuokuang Road, Taichung 40227, Taiwan

**Keywords:** total leaf area, analysis of variance, *Phalaenopsis*, growth characteristics

## Abstract

*Phalaenopsis* orchids are highly economical ornamental potted plants. Controlling their production schedule requires information on the leaf development characteristics of the orchids. *Phalaenopsis* leaves affect the plant’s photosynthesis, respiration, and transpiration. The leaf growth conditions can serve as a development index for greenhouse management. The use of the growth characteristics of *Phalaenopsis* leaves as the basis for greenhouse cultivation and management needs to be studied. The allometry of *Phalaenopsis* leaves is worth studying. The goal of this research was to investigate the allometry of *Phalaenopsis* leaves and develop prediction models of the total leaf area. Then, these total leaf area models were developed and validated. In this study, five *Phalaenopsis* varieties (amabilis, Sin-Yuan beauty, Ruey Lish beauty, Ishin KHM1095, and Sogo F1091) were selected. Each sample had five mature leaves. The lengths, widths, and areas of the sequential leaves were measured, and then the length ratios, width ratios, and area ratios were calculated. The top and bottom models were used to calculate the total leaf areas. The results indicate that no significant differences could be found in the length ratios, width ratios, and area ratios of the sequential leaves from the same variety. However, significant differences were found in these leaf characteristics between different varieties. The observation of leaf growth characteristics can be used to provide useful information for *Phalaenopsis* management. Comparing the predictive criteria of the two models, the top model had a better predictive ability than the bottom model. From a practical viewpoint, measuring the top leaf area is easier than measuring the bottom leaf area in a greenhouse operation. Comparing the effects of the sample numbers on the predictive ability of the model, the sample number of 30 was sufficient to ensure the accuracy of the total leaf area measurements. We provide an easy and accurate method to measure the total leaf area of *Phalaenopsis*. The calculated values of total leaf areas can be incorporated into decision models for smart management.

## 1. Introduction

Moth orchids (*Phalaenopsis* spp.) are high-value potted ornamental plants. In the orchid industry, the special characteristics of *Phalaenopsis* are shorter juvenile periods, rapid growth, and easy-to-control spiking and flowering. These orchids have stylish and exotic shapes, various sizes and colors of blossoms, and long vase lives. Their shape, length, width, and area are affected by environmental factors (day and night temperatures) and fertilization (fertilizer concentration and formulation). The timing of the potted flowers being sent to the market determines the market price [1,2]. To ensure the spiking and flowering period, affecting factors such as temperature and light intensity are controlled in a modern greenhouse [3,4]. In the study by Paradiso and De Pascale [4], they emphasized that three factors, leaf size, temperature, and light intensity, are the key factors in the flowering quality of *Phalaenopsis*. That is, the maturation conditions of the plants affect the size, number, and bright color of the flowers. The growth conditions of the leaves need to be investigated. However, quantified leaf characteristics are lacking for the management operation of an orchid greenhouse.

The term allometry is used to describe the scaling relationship between the size of a part of a body and the size of the whole body. The typical application of allometry is in using the leaf width or length to predict its area. In this study, the concept of the allometry approach was proposed to study the growth characteristics of *Phalaenopsis* leaves.

In a detailed review, Lemaire et al. [5] introduce evidence of allometry between metabolic and structural mass, and the concentrations of nutrient also have a relationship with biomass. Shi et al. [6] developed novel models to describe the relationship between leaf shape and other variables for fifteen vine species. Fanourakis et al. [7] proposed eight empirical linear equations to describe the relationship between leaf area and length, width, and the product of length and width. Colchado-Lopez et al. [8] introduced the rhizochron index model to describe lateral root branching and elongation patterns in the root architecture. Feng et al. [9] adopted the concept of allometry to observe the relationship between vegetative and reproductive traits, such as peduncles and flowers, inflorescence length and leaf area, and total floral area and total leaf area, in different species of orchids. Mantovan [10] studied the relationship between plant size and dry mass partitioning in the epiphytic orchid *Lankesterella ceracifolia*, and several allometric models were validated.

A crop model needs to consider leaf phenology [11]. The relationship between the carbohydrate contents in leaves and inflorescence initiation in *Phalaenopsis* has been observed [12]. The development of a leaf can serve as an index of growth stages [13]. The number and quality of leaves can be illustrated to serve as an index for greenhouse management [3]. Recently, with the development of the camera technique, pictures of orchid leaves can be easily collected and analyzed [14]. To the best of the authors’ knowledge, no quantified index has been proposed to represent the growing conditions of leaves for *Phalaenopsis.*

To measure a leaf area continuously, a non-destructive estimation of the leaf area (A) with the leaf length (L) and width (W) is of interest to researchers [15,16,17,18]. Empirical equations relating the length and width to the area have been developed, such as for corn, grapes, muskmelons, and *capsicum* [19]. Swart [20] reported six empirical models and found the best equation was A = c_1_ (L × W) + c_2_ (L × W)^2^. In this equation, L, W, and A are the length, width, and area of a capsicum leaf, and c_1_, c_2,_ and the following c_i_ are constants. Shi et al. [21] studied the leaf area equations of four types of special leaf shapes of trees and found that the Montgomery equation could be used for all special plants. This equation is A = c_3_ (L × W). In a study on the leaf area equations of six Magnoliaceae species, He et al. [22] proposed that the Montgomery equation could provide a standard measurement for leaf morphology. Mazzini et al. [23] studied the leaf area models of six genotypes of citrus and proposed a linear equation with an intercept and slope, A = c_4_ L × W + c_5_, which could be applied to other citrus genotypes. Yu et al. [24] examined the estimation equations of 15 species of vines with different leaf shapes and recommended the Montgomery equation. Chen and Lin [25] studied the leaf area equations of *Phalaenopsis* leaves, and four popular cultivars in Taiwan were used to measure their leaf areas, lengths, and widths. The proposed equation, A = 0.725 L × W, was presented as the adequate equation.

The total leaf area is a very important index for plant growth. It involves photosynthesis, respiration, and transpiration. A total leaf model can be developed with the concept of allometry. The development of total leaf area models has been proposed using the following equations.

The calculation models of the total leaf areas of a shoot were reviewed by Koyama and Smith [19]. The total leaf area (Atot) of a shoot is related to the sum of a single leaf width (ΣWidth) and the maximum leaf length (Lmax).

Atot = c_6_ ΣWidth × Lmax (1)

Ogawa et al. [26] proposed the following empirical equations:

Atot = c_7_ (Lmax)^c8^ (2)

Sun et al. [27,28] found that the Atot is related to the product of the maximum leaf area (Amax) of a shoot and the total number of leaves on each shoot (N). Their model is as follows:

Atot = c_9_ N × (Amax) (3)

Koyama et al. [29] arranged the affecting factors and proposed the following prediction equation of the total leaf area with the total number of leaves (N):

Atot = c_10_ × N^C11^ (4)

Teobaldelli et al. [30] proposed an empirical model of the total leaf area:

Atot = c_12_ N^C13^ W_max_ (5)

where W_max_ is the maximum width of the leaves.

Lopes and Pinto [31] studied the formula of the total leaf area for each shoot of a wine grape vine and found the total leaf area of a shoot can be estimated as the mean of the maximum (MaxArea) and minimum leaf area (MinArea):

Atot = C_14_ × N × (MaxArea + MinArea)/2 (6)

These models were validated via the observation of the linear equation between the predicted values and actual values of the total leaf areas of a shoot. All leaves are assumed to have the same shape and size.

A lot of plants have many shoots and leaves. *Phalaenopsis* is an epiphytic and monopodia orchid. The plant structure has very short internodes and succulent-like leaves. A growth characteristic of *Phalaenopsis* is an enlarged leaf phenomenon. That is, the leaves are developed sequentially. In the leaf growth of *Phalaenopsis*, according to the observations of growers, the newly grown leaves are larger than the previous leaves.

The previous models in the literature that assume that all leaves have a similar shape and area could not be used for *Phalaenopsis.* That is, these proposed leaf area equations in the literature cannot be directly applied to *Phalaenopsis*.

For managing *Phalaenopsis* production, the information on leaf shape, length, width, and total leaf area is a very useful index. It is easily analyzed in photo monitoring systems. In this study, the leaf characteristics and allometry of leaves were quantified for the leaf development characteristics of *Phalaenopsis.* The prediction equations of the total leaf area were developed and validated. This study could provide basic information for greenhouse management.

## 2. Results

### 2.1. The Leaf Specification

#### 2.1.1. The Ratio Characteristics

The statistics for the total leaf areas of the five *Phalaenopsis* varieties are listed in Table 1. Comparing the mean areas, amabilis has the smallest value, and Ishin KHM 1095 has the largest.

**Figure 1:**
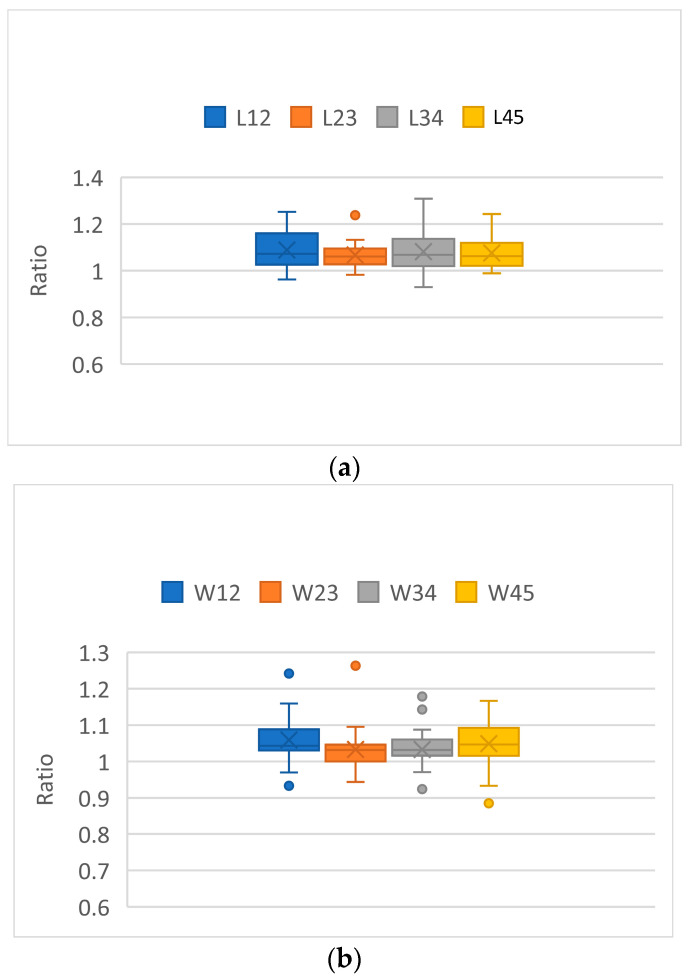

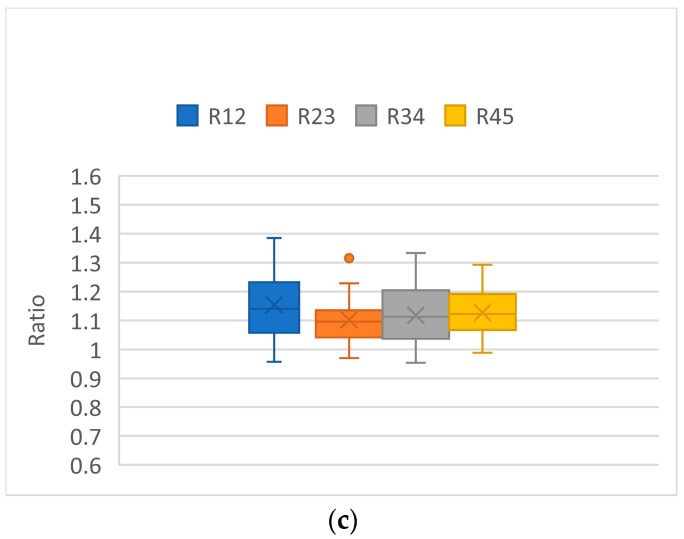
The leaf length, width, and area ratios for amabilis. No significant differences could be found. There are no significant differences between the four ratios in the different leaves. (**a**) Length ratios (L12 is the ratio between no. 1 leaf length to no. 2 leaf length, etc.). (**b**) Width ratios (W12 is the ratio between no. 1 leaf width to no. 2 leaf width, etc.). (**c**) Area ratios (R12 is the ratio between no. 1 leaf area to no. 2 leaf area, etc.). The colored circles showed some extreme measurement values.

Figure 1 shows the leaf length, width, and area ratios for amabilis. The results of the analysis of variance (ANOVA) are insignificantly different for the length ratio (F (3, 120) = 0.502; *p* = 0.682), width ratio (F (3, 120) = 1.546; *p* = 0.206), and area ratio (F (3, 120) = 1.579; *p* = 0.198). There were 5 mature leaves for *Phalaenopsis* in the 12 cm pot. The length ratios of L_1_ to L_2_, L_2_ to L_3_, L_3_ to L_4_, and L_4_ to L_5_ were similar, and no significant differences could be found. The same results were found for the width ratios and area ratios. That is, the W ratios of W_1_ to W_2_, W_2_ to W_3_, W_3_ to W_4_, and W_4_ to W_5_ were similar, and no significant differences could be found. The same conclusion was found for the area ratios. The area ratios of A_1_ to A_2_, A_2_ to A_3_, A_3_ to A_4_, and A_4_ to A_5_ were similar, and no significant differences could be found. The length, width, and area ratios in sequence leaves are useful characteristics of *Phalaenopsis*. The leaves are developed sequentially in *Phalaenopsis*, whereby the newly grown leaves are larger than the previous leaves. The results for *Phalaenopsis* amabilis confirmed this leaf growth characteristic.

The analysis of variance results for Sin Yuan indicated no significant differences between the length ratios (F (3, 120) = 1.970; *p* = 0.124), width ratios (F (3, 120 = 0.497; *p* = 0.685), and area ratios (F (3, 120) = 2.217; *p* = 0.092).

The evaluation of the length ratios, width ratios, and area ratios for Rey Lish was performed with a one-way ANOVA. The results of the ANOVA were insignificantly different for the length ratios (F (3, 120) = 0.655, *p* = 0.582), width ratios (F (3, 120) = 2.111, *p* = 0.983), and area ratios (F (3, 120) = 0.918; *p* = 2.683).

To evaluate the leaf ratio of Ishin KHM1095, a one-way ANOVA was performed. The results revealed no significant differences between the four leaf ratios (F (3, 120) = 2.260; *p* = 0.085), width ratios (F (3, 120) = 2.070; *p* = 0.108), and area ratios (F (3, 120) = 2.393; *p* = 0.720).

For the Sogo F1091 variety, the results of the one-way ANOVA showed the effect of the ratios of the characteristics was insignificant. The results were F (3, 120) = 1.633, *p* = 0.185, for the length ratios; F (3, 120) = 2.113, *p* = 0.0781, for the width ratios; and F (3, 120) = 1.211, *p* = 0.309, for the area ratios.

According to the observations of growers, the leaves are developed sequentially in *Phalaenopsis*, such that the newly grown leaves are larger than the previous leaves. The results for the five varieties of *Phalaenopsis* confirm this leaf growth characteristic. This provides a quantitative basis for the leaf characteristics of *Phalaenopsis.*

#### 2.1.2. The Ratio Characteristics of Five Varieties

In this study, the leaf characteristics of five *Phalaenopsis* varieties were measured.

In the above discussion, the length ratio, width ratio, and area ratio were insignificant for each variety. Then, all the data for each variety were pooled for further analysis. For example, the L_12_, L_13_, L_34_, and L_45_ data of a variety were pooled into a data set.

**Figure 2:**
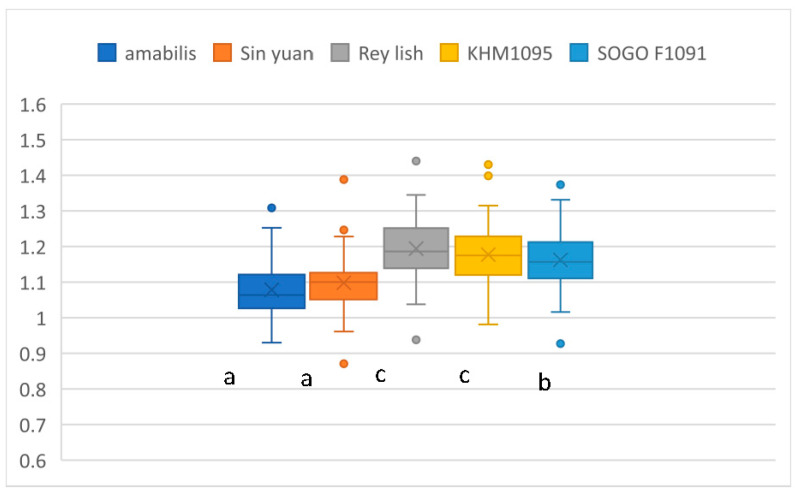
The ratios of leaf length for five *Phalaenopsis* varieties. Means followed by the same letter are not significantly different (*p* < 0.05; Tukey’s multiple comparison tests). The letters a, b, and c represent whether there is a significant difference. The colored circles showed some extreme measurement values.

A comparison of the length ratios for the five varieties is shown in Figure 2. The results of the ANOVA are significantly different (F (4, 615) = 50.150; *p* < 0.001). In multiple comparisons, Tukey’s tests are significantly different for the 3 groups at a 95% confidence level. The order for the average length ratios is Rey Lish and KHM1091 > Sogo F1091 > amabilis and Sin Yuan.

**Figure 3:**
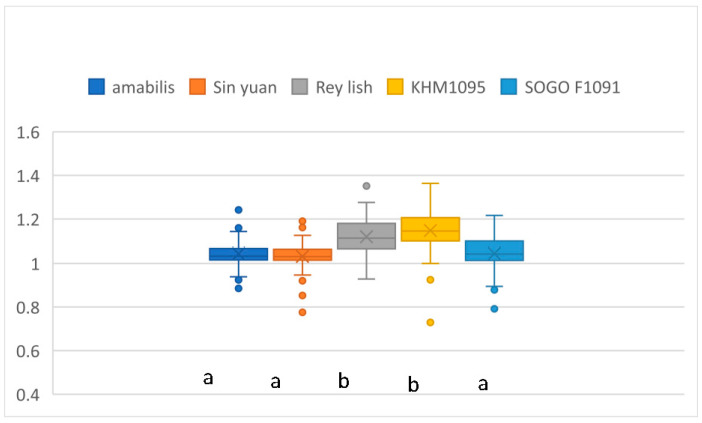
The ratios of leaf width for five *Phalaenopsis* varieties. Means followed by the same letter are not significantly different (*p* < 0.05; Tukey’s multiple comparison tests). The letters a, b, and c represent whether there is a significant difference. The colored circles showed some extreme measurement values.

A comparison of the width ratios for the five varieties is shown in Figure 3. The results of the analysis of variance show a significant difference (F (4, 615) = 53.113; *p* < 0.001). For multiple comparisons, Tukey’s tests are significantly different for the 2 groups at a 95% confidence level. The order for the average width ratios is Rey Lish and KHM1091 > amabilis and Sin Yuan and Sogo F1091.

**Figure 4:**
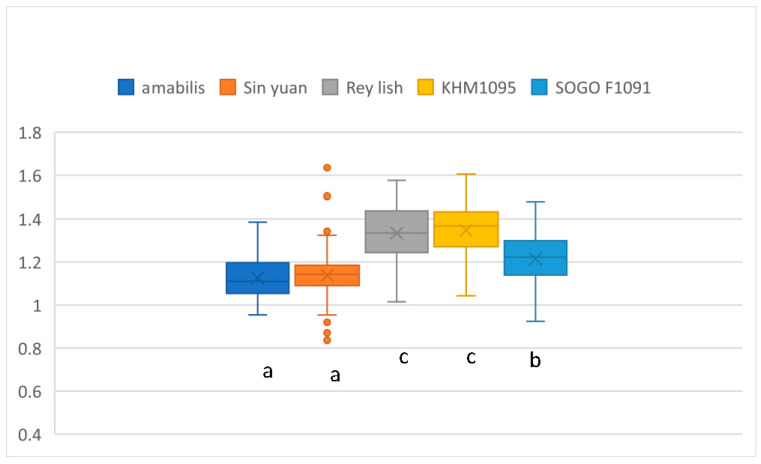
The area ratios for five *Phalaenopsis* varieties. Means followed by the same letter are not significantly different (*p* < 0.05; Tukey’s multiple comparison tests). The letters a, b, and c represent whether there is a significant difference. The colored circles showed some extreme measurement values.

A comparison of the area ratios for the five varieties is shown in Figure 4. The results of the ANOVA indicate a significant difference (F (4, 615) = 101.74; *p* < 0.001). For the multiple comparisons, Tukey’s test measurements show significant differences between the 3 groups at a 95% confidence level. The order for the average area ratios is Rey Lish and KHM1095 > Sogo F1091 > amabilis and Sin Yuan.

In Table 1, the flower styles and the plant heights of amabilis and Sin Yuan differ. However, both varieties had similar leaf ratio characteristics. The Rey Lish and KHM1095 varieties have big red flowers and similar plant heights. They have similar leaf ratio characteristics. Sogo F1091 has a similar plant height and flower size to amabilis and Sin Yuan. The flower color is different. The leaf ratio characteristics differ from those of other varieties. Due to the diversity of the *Phalaenopsis* varieties, it is necessary to establish each variety’s specific leaf ratio characteristics.

Growers have observed the sequential development of *Phalaenopsis* leaves. The special characteristic of *Phalaenopsis* is that the areas of newly grown leaves are larger than those of previous leaves. The results for the five varieties of *Phalaenopsis* reveal that each variety has a special growth characteristic. The basic leaf characteristics of each variety need to be established.

### 2.2. The Prediction Performance for the Total Leaf Area

The mean values of the area ratios for the five varieties were used in the top and bottom models to predict the total leaf areas. The prediction error is defined as the predicted value minus the actual measured total leaf area value. The prediction errors for the two models of the five varieties are listed in Table 2.

For the criterion emin, the top model has smaller emin values than those of the bottom model, except for Rey Lish. All the emax values of the top model are smaller than those of the bottom model. For the criterion |e|ave, the average mean absolute error, the top model has smaller values than those of the bottom model. The |e|ave values of the top model are 19.15, 23.33, 32.48, 22.25, and 30.79 for the five varieties, respectively. However, the |e|ave values of the bottom model are 21.13, 24.19, 48.15, 45.94, and 32.91 for the same varieties, respectively. Comparing these predictive criteria, the top model has a better predictive performance than the bottom model.

**Figure 5:**
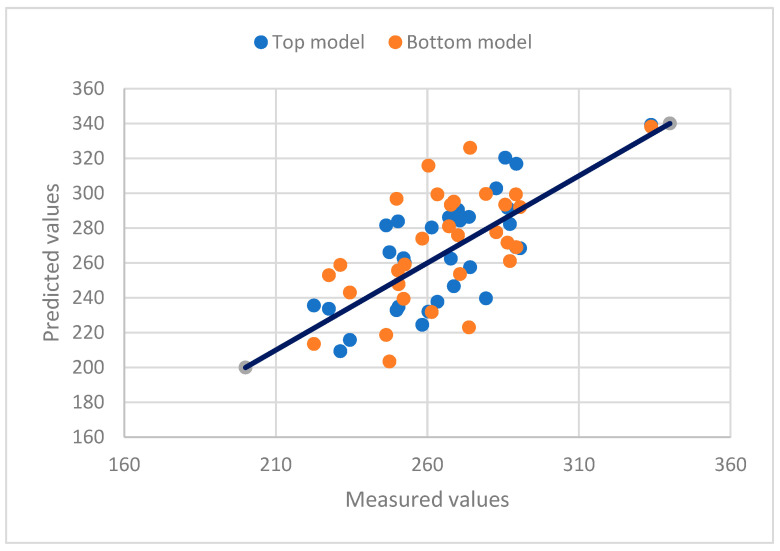
The relationship between the measured values and the predicted total leaf areas using the top and bottom models for amabilis. A solid line represents a 1:1 relationship. The correlation analysis result between the top model values and the measured values is 0.9968 (*p* < 0.001), and the correlation analysis result between the bottom model values and the measured values is 0.9955 (*p* < 0.001).

**Figure 6:**
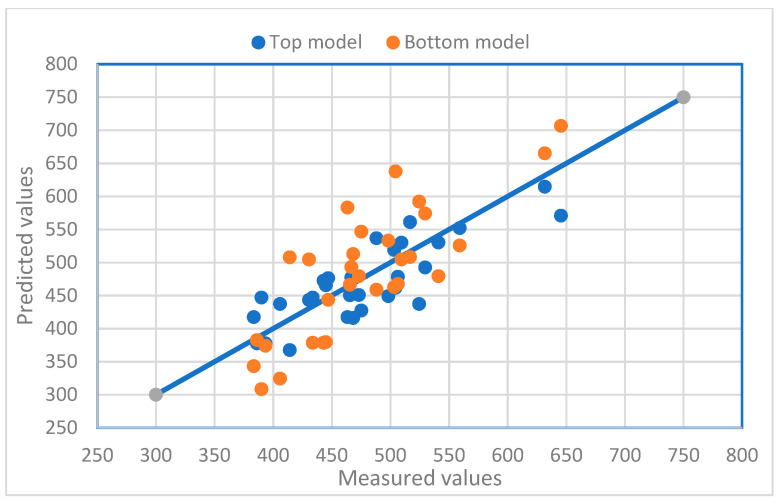
The relationship between the measured values and the predicted total leaf areas using the top and bottom models for Rey Lish. A solid line represents a 1:1 relationship. The correlation analysis result between the top model values and the measured values is 0.9970 (*p* < 0.001), and the correlation analysis result between the bottom model values and the measured values is 0.9931 (*p* < 0.001).

**Figure 7:**
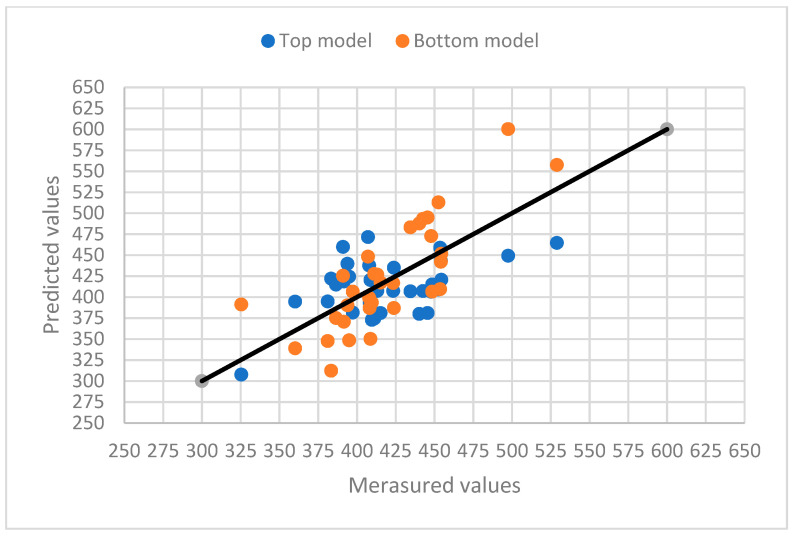
The relationship between the measured values and the predicted total leaf areas using the top and bottom models for Sogo F1091. A solid line represents a 1:1 relationship. The correlation analysis result between the top model values and the measured values is 0.9962 (*p* < 0.001), and the correlation analysis result between the bottom model values and the measured values is 0.9956 (*p* < 0.001).

The relationships between the measured values and the predicted total leaf areas using the two models for the three varieties are presented in Figure 5, Figure 6 and Figure 7. The diversified scatter points along the X = Y lines reveal the prediction problems of the models. If only a few samples are used for leaf area measurements, the predicted total leaf area may not be accurate enough. 

The effects of the sample numbers on the prediction performance for the total leaf area are listed in Table 3. A sample number of 10 means 10 plants were used to measure their top or bottom areas. Then, the total leaf areas were calculated with the top model or the bottom model, The mean error percentages of the top model are 5.4% (amabilis), 1.3% (Sin Yuan), −5.9% (Rey Lish), −5.1% (KHM 1095), and −4.0% (Sogo F1091). The mean error percentages of the bottom model are −2.9% (amabilis), −2.6% (Sin Yuan), 3.7% (Rey Lish), 6.7% (KHM 1095), and 2.3% (Sogo F1091). The mean error percentages of the two models are > 2%, except for the top model for Sin Yuan.

A sample number of 20 means 20 plants were used to measure their top and bottom leaf areas and then to calculate their total leaf areas. The error percentages are reduced. With a sample number of 30, 30 orchids were used to measure their top and bottom leaf areas. The total leaf areas were calculated. The mean error percentages of the top model are 0.4% (amabilis), −0.3% (Sin Yuan), −1.7% (Rey Lish), 0.8% (KHM 1095), and 1.2% (Sogo F1091). The mean error percentages of the bottom model are 1.7% (amabilis), −0.8% (Sin Yuan), 1.3% (Rey Lish), −1.1% (KHM 1095), and 1.0% (Sogo F1091). The mean error percentages of the two models are < 2%. In a study on the allometry of the epiphytic orchid *Lankesterella ceracifolia* [10], the sample number was thirty-five. This number exceeds the investigated number in our study. Based on the results of this study, a sample number of thirty is enough. That is, 30 leaves are measured for each quality control work. This technique could cooperate with the standard operating procedure (SOP) of greenhouse management.

## 3. Discussion

In the cultivation of *Phalaenopsis* [3], Anthura b.v. illustrates mature orchids with their shapes and number of leaves. However, the study still lacks a quantified criterion. With the concept of allometry, growth characteristics can be evaluated with the measurement of leaf length, width, and area. The development of leaf shape is affected by the environment (temperature, light intensity, etc.) and fertilization. The observation of leaf growth characteristics provides useful information on *Phalaenopsis* management. For example, if the leaf measurement data during a period of quality control work indicated that the ratios of length, width, and area were different from the previous standard of this variety, the management of microclimate factors and the fertilization technique would need to be modified.

The total leaf area of an orchid is important information to evaluate its mature state. The total leaf area can be calculated with the measurement of the top leaf area and the model developed in this study. Then, the total leaf area could serve as a useful index.

From a practical viewpoint, measuring the top leaf area is easier than measuring the bottom leaf area. To increase production capacity, orchid pots are very densely placed in commercial greenhouses. The distribution of the upper leaves retards the measurement of the lowest leaf area.

Recently, drones have been applied in greenhouses to obtain crop information with cameras. A lot of pictures of plants could be captured within a short time. Many data on the top area of orchids were easily collected [32]. The information on the total leaf area can then be calculated with the top model developed in this study. This study provides an easy and smart method to enhance the production of *Phalaenopsis*. When the total leaf area is calculated, this parameter can be used for crop models to evaluate growth conditions and manage other crops.

To the authors’ knowledge, this study is the first to research total leaf area models for orchids. Lawless et al. [11] proposed a wheat canopy model that links leaf area and its phenology. In this study, the total leaf area was introduced and validated. This model can be incorporated into these crop models to enhance their predictive performance. Lee et al. [12] found a correlation between carbohydrate concentrations and the soluble sugars in the leaves of *Phalaenopsis*. They present the basic concept of the relationship between the total leaf area and the quantity and quality of flowers. Further studies need to be performed. Paradiso et al. [1] introduced the effect of plant size on the flowering of *Phalaenopsis*. However, their definition of plant size was not precise enough. The values of the total leaf area developed in this study could provide a more precise index for plant size. Crop models have been proposed and emphasized by researchers [16,28,33,34]. In their models, the total leaf area involves photosynthesis, respiration, and transpiration. However, measuring each leaf area and calculating the total leaf area is time-consuming work in the applications of these crop models. The model developed in this study provides an easy way to calculate the total leaf area.

In a study on the vegetative traits and flowering quality of *Phalaenopsis* for different genotypes, van Tongerlo et al. [35] found the after-effects of treatments applied during vegetative growth on flowering traits and plant biomass, and the number of leaves was positively correlated with the flowering quality. The authors mention that the measurements of the plant biomass and number of leaves can be used as predictors of flowering capacity and quality. The dry mass of *Phalaenopsis* is a function of the length and width [25]. In this study, besides the number of leaves, the shape, length, width, and total area are growth characteristics that could serve as quality control work for the cultivation of *Phalaenopsis.*

In this study, *Phalaenopsis* was selected as the research object. The nursery of these orchids was grown with the mericlone tissue culture technique. These genetic characteristics can supply a uniform nursery. The orchids were cultivated in a greenhouse with modern equipment. The aerial environment (temperature, humidity, light intensity, and air velocity) and root environment (moisture content and EC in the substrate) were all controlled in uniform conditions. In this study, the leaf ratio characteristics were established. Because of the uniform growth characteristics of *Phalaenopsis*, the variations in leaf dimensions in Table 2 were limited. The accuracy of the calculation of the total leaf area with the top model developed in this study is very helpful.

However, a nursery of other crops may be planted with seeds or cutting propagation materials. The aerial and root environment in an open field or simple protected culture cannot be a uniform microclimate such as in a modern greenhouse with all kinds of equipment. It may not be possible to adopt the technique developed in this study for all crops. The suitability of the developed model in this study needs to be further studied for other crops.

## 4. Materials and Methods

### 4.1. The Total Leaf Area Model

**Figure 8:**
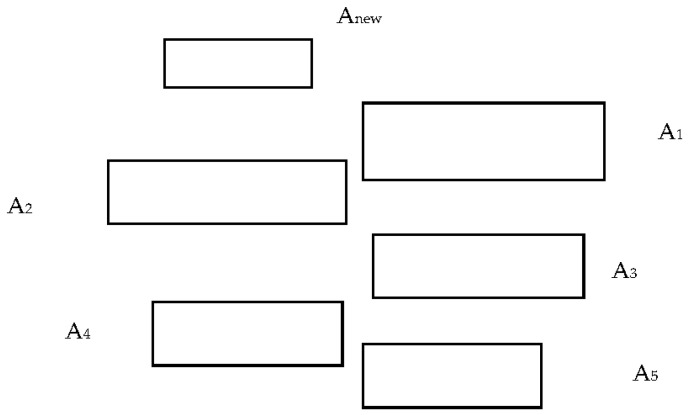
Diagram of *Phalaenopsis* leaves.

*Phalaenopsis* orchids with 5 leaves planted in a 12 cm pot were used as the test materials. The notation of the leaf number was adopted from the study by Hew and Yong [4]. The areas of new immature leaves and cotyledons were not considered in this study. The diagram of the number of leaves is shown in Figure 8. A_new_ is a new immature leaf. A_1_ to A_5_ are the sequential numbers of leaves. A_1_ is the top leaf, and A_5_ is the bottom leaf. The cotyledon is not presented in the figure.

The bottom leaf is called the fifth leaf, and the area (A_5_) is a function of the leaf length (L_5_) and width (W_5_).

A5 = K (L_5_ × W_5_) (7)

In this study, K = 0.725 [25].

For the next leaf above the bottom leaf, the length (L_4_) and width (W_4_) of the fourth leaf (the L_4_) have a ratio relation with the bottom leaf.

L_4_ = aL_5_ (8)

W_4_ = bW_5_ (9)

The ratio of the length of the fourth leaf to the length of the fifth leaf is denoted as L_45_. The ratio of the width of the fourth leaf to the width of the fifth leaf is denoted as W_45_. The ratio of the area of the fourth leaf to the area of the fifth leaf is denoted as R_45_, and so on for other notations.

Therefore, the relationship between the leaf area of the fourth leaf (A_4_) and the area of the bottom leaf (A_5_) is expressed as

A_4_ = K (L_4_ × W_4_) = K × a × b × (L_5_W_5_) (10)

It is assumed the ratio between L_3_ and L_4_ has the same value as a, and the ratio between W_3_ and W_4_ has the same value as b.

The area of the next upper leaf, the third leaf (A_3_), can be derived as

A_3_ = K (L_3_ × W_3_) = K × a × b (L_4_W_4_) = Ka^2^b^2^ (L_5_W_5_) (11)

With the same procedure,

A_2_ = Ka^3^b^3^ (L_5_W_5_) (12)

The area of the top leaf, the first leaf, can be calculated as

A_1_ = Ka^4^b^4^ (L_5_W_5_) (13)

In a 4-inch pot of *Phalaenopsis*, there are 5 mature leaves. The total leaf area of a *Phalaenopsis* orchids with five leaves is

Atot = A_5_ + A_4_ + A_3_ + A_2_ + A_1_ = K (L_5_W_5_) (1 + ab + a^2^b^2^ + a^3^b^3^ + a^4^b^4^) (14)

This equation is called the bottom model. That is, the total leaf area is calculated with the bottom leaf area.

If the top leaf (A_1_) serves as the basic leaf to calculate the total leaf area,

(15) $$\mathrm{Atot}=K\left(L_{1}W_{1}\right)\left(1+\frac{1}{\mathrm{ab}}+{\left(\frac{1}{\mathrm{ab}}\right)}^{2}+{\left(\frac{1}{\mathrm{ab}}\right)}^{3}+{\left(\frac{1}{\mathrm{ab}}\right)}^{4}\right)$$

This equation is called the top model.

### 4.2. Testing Materials

This experiment was performed in a commercial orchid nursery, Taida Orchids Company (Dacun, Changhua county, Taiwan). Five commercial varieties were studied. These were amabilis, *Dtps.* Sin-Yuan beauty (Sin-Yuan), *Phal.* Ruey Lish beauty (Ruey Lish), Ishin KHM1095 (KHM-1095), and the Sogo Co. Ltd. F1091 variety (Sogo F1091). All orchids were cultivated in 12 cm pots and had at least 5 mature leaves. The plant and flower styles of these *Phalaenopsis* varieties are listed in Table 4. There were thirty-one samples for each variety. Each sample had five mature leaves.

### 4.3. Measurement of Leaf Characteristics

Before starting the measurements, the leaves were detached from the plants. The leaf and maximum widths were measured with a digital vernier caliper (CARMA, Taipei, Taiwan). The resolution was 0.01 mm, and the accuracy was 0.1 mm. The leaf areas were measured with a Li-300A area meter (LI-COR, Inc., Lincoln, NE, USA). The resolution of this meter was 1 min^2^. The accuracy was 1% for samples smaller than 50 cm^2^ after calibrating.

### 4.4. Data Analysis

This experiment used Sigmaplot version 13.0 (SPSS Inc., Chicago, IL, USA) and EXCEL 2007 (Microsoft^®^ Office Excel 2007) for statistical analyses. For each leaf characteristic in each treatment, an analysis of variance was used to test whether there were significant differences between the various positions or varieties.

If a significant difference was found, post hoc tests were used to determine the differences between the treatments.

Before the ANOVA tests, a homogeneous test was performed to test whether the amount of variation between the treatments was equal. If the variance was equal, Tukey’s test was used for the post hoc test.

### 4.5. Model Evaluation

To evaluate the predictive abilities of the two models, the predicted total leaf area values of the models and the actual measured values were compared. The difference between the predicted value of a model and the measured value is termed the error. The criteria for comparison were the maximum error, emax; the minimum error, emin; and the average sum of absolute errors, |e|ave.

*ei* = predictive value–measured value (16)

(17) |e|ave=∑|ei|/n

## 5. Conclusions

In this study, the lengths, widths, and areas of *Phalaenopsis* leaves were measured to study the growth characteristics. The allometry of the total leaf area was modeled and validated. Five *Phalaenopsis* varieties were used. All samples had five mature leaves excluding the cotyledons. Sequential leaves’ lengths, widths, and areas were measured, and then the length, width, and area ratios were calculated. Two models, the top and bottom models, were developed to calculate the total leaf areas using the top leaf area or bottom leaf area as the parameter.

The results indicate no significant differences could be found for the length ratios, width ratios, and area ratios in the same variety. However, significant differences were found for these leaf characteristics between different varieties. The growth characteristics could be evaluated with the measurement of the leaf lengths, widths, and areas. The observation of leaf growth characteristics could be used to evaluate the growth of *Phalaenopsis*. The measurement data obtained during a period of quality control work could be compared with the previous standard values of this variety. The management of microclimate factors and fertilization techniques could be modified if necessary.

Compared with the predictive criteria of the two models, the top model calculated with the top leaf area has a better predictive ability than that of the bottom model calculated with the lowest leaf area. From a practical viewpoint, the top leaf area is easier to measure. The sample number of thirty leaves is reasonable to ensure the accuracy of the total leaf area measurement. With modern sensing techniques, the top areas of many *Phalaenopsis* leaves can be measured easily. The total leaf areas of orchids can be calculated easily. This index could then be used for greenhouse management. This technique could be employed in the standard operating procedure (SOP) of greenhouse management.

Future studies could include validating this total leaf model for more varieties of *Phalaenopsis* and other crops. This total leaf area model will be incorporated into the decision model for a study on smart orchid production. The relationship between the total leaf area and the quantity and quality of flowers in *Phalaenopsis* will be studied to ensure the applicability of this total leaf area model.

## Figures and Tables

**Table 1:** Statistics for the total leaf areas of five *Phalaenopsis* varieties.

	Amabilis	Sin Yuan	Rey Lish	KHM1095	Sogo F1091
Mean	265.34	414.56	478.04	577.61	419.82
S.D.	4.11	7.72	11.84	13.06	6.86
Minimum	222.59	329.52	383.42	429.15	325.41
Maximum	333.85	478.73	645.53	734.36	528.95

S.D.: standard deviation.

**Table 2:** Statistical results of the prediction errors for two total leaf area models of five varieties.

	Amabilis	Sin Yuan	Rey Lish	KHM 1095	Sogo F1091
Top model					
$\;\;\;e_{min}$	−39.68	−98.01	−87.22	−66.3	−64.47
$\;\;\;e_{max}$	35.07	35.18	57.05	37.54	68.84
|e|ave	19.15	23.33	32.48	22.25	30.79
Bottom model					
$e_{min}$	−50.74	−128.1	−81.52	−107.05	−71.15
$\;e_{max}$	55.44	65.5	133.51	106.68	102.71
|e|ave	21.13	24.19	48.15	45.94	32.91

**Table 3:** Effects of test numbers of samples on the prediction performance for total leaf area.

		Amabilis	Sin Yuan	Rey Lish	KHM 1095	Sogo F1091
N = 10	Area	277.92 (100%)	415.97	505.51	569.62	419.03
	Top model	292.99 (5.4%)	421.38 (1.3%)	474.93 (−5.9%)	540.69 (−5.1%)	402.80 (−4.0%)
	Bottom model	269.88 (−2.9%)	405.15 (−2.6%)	524.2 (3.7%)	607.53 (6.7%)	428.74 (2.3%)
N = 20	Area	274.83 (100%)	414.56	480.20	574.14	410.77
	Top model	279.83 (1.8%)	417.88 (0.8%)	470.80 (−1.9%)	559.17 (−2.6%)	404.40 (−1.6%)
	Bottom model	270.08 (−1.7%)	407.10 (−1.8%)	483.13 (0.6%)	583.95 (1.7%)	414.5 (0.9%)
N = 30	Area	265.34 (100%)	413.14	478.04	577.60	419.82
	Top model	260.61 (0.4%)	411.95 (−0.3%)	470.08 (−1.7%)	582.33 (0.8%)	414.60 (−1.2%)
	Bottom model	269.94 (1.7%)	409.66 (−0.8%)	484.10 (1.3%)	570.93 (−1.1%)	424.14 (1.0%)

**Table 4:** Plant and flower styles of the five *Phalaenopsis* orchid varieties.

	Amabilis	Sin Yuan	Rey Lish	KHM1095	Sogo F-1091
Plant height (cm)	40	50	70	70	40
Flower size (cm)	4.5	7.5	10.5	10.0	4.5
Flower color	White	Yellow with red lip	Red	Red	Pink

## Data Availability

Not applicable.
